# Integrated Macrogenomics and Metabolomics Explore Alterations and Correlation between Gut Microbiota and Serum Metabolites in Adult Epileptic Patients: A Pilot Study

**DOI:** 10.3390/microorganisms11112628

**Published:** 2023-10-25

**Authors:** Kaiping Zhou, Lijing Jia, Zhuofeng Mao, Peipei Si, Can Sun, Zhenzhen Qu, Weiping Wang

**Affiliations:** 1 Key Laboratory of Neurology of Hebei Province, Department of Neurology, The Second Hospital of Hebei Medical University, Shijiazhuang 050004, China; 2 Department of Neurology, Peking University Third Hospital, Beijing 100080, China

**Keywords:** epilepsy, gut microbiota, serum metabolites, multiomic analysis

## Abstract

Epilepsy (EP) is a complex brain disorder showing a lot of unknows reasons. Recent studies showed that gut microbiota can influence epilepsy via the brain–gut axis. Nevertheless, the mechanism by which gut microbiota affects adult epilepsy still remains unclear. In this study, fecal and serum samples were obtained from patients with epilepsy and normal controls. Using an integrated analysis, sequencing was performed by macrogenomics and high-throughput targeted metabolomics with various bioinformatics approaches. The macrogenomic sequencing revealed significant changes in microbial structure in patients suffering from epilepsy. For example, at the phylum level, the relative abundance of Actinobacteria, Bacteroidetes and Proteobacteria showed an increase in the patients with epilepsy, whereas that of Firmicutes decreased. In addition, the patients with epilepsy had significantly differential metabolite profiles compared to normal controls, and five clusters with 21 metabolites, mainly containing the upregulation of some fatty acids and downregulation of some amino acids. Tryptophan (AUC = 91.81, *p* < 0.0001), kynurenine (AUC = 79.09, *p* < 0.01) and 7Z,10Z,13Z,16Z-Docosatetraenoic acid (AUC = 80.95, *p* < 0.01) may be used as potential diagnostic markers for epilepsy. Differential serum metabolites have effects on tryptophan metabolism, iron death and other pathways. Furthermore, a multiomic joint analysis observed a statistically significant correlation between the differential flora and the differential serum metabolites. In our findings, a macrogenomic analysis revealed the presence of dysregulated intestinal flora species and function in adult epileptic patients. Deeper metabolomic analyses revealed differences in serum metabolites between patients with epilepsy and healthy populations. Meanwhile, the multiomic combination showed connection between the gut microbes and circulating metabolites in the EP patients, which may be potential therapeutic targets.

## 1. Introduction

Epilepsy is a prevalent central nervous system illness with roughly 5 million new patients diagnosed each year [1]. The etiological mechanism of epilepsy is complex and diverse, including abnormal brain structure, genetic abnormalities, causes of infection, as well as metabolic and immune factors. In many patients, the reason for epilepsy remains unknown [2]. Pharmacotherapy is the primary curative treatment for epilepsy. Nevertheless, around 30–40% of patients are hypersensitive to antiepileptic drugs and are susceptible to drug resistance [3]. Therefore, it is crucial to understand the etiopathogenesis of epilepsy in more detail as well as to identify new and more effective strategies for preventing and treating epilepsy.

Gut microbiota (GM) are microorganisms living in the digestive tract, with the majority as bacteria, archaea, fungi and microeukaryotes [4]. The set of all intestinal microorganism genes represents a genetic repertoire. This can be regarded as two orders of magnitude higher when compared with that of the human genome [5]. To a certain degree, the GM can also be regarded to be an ‘essential organ’ of the human body [6]. Under the dynamic equilibrium state, the GM is symbiotic with the host and supports the normal physiological processes. The structure and function of the intestinal flora will alter as well as result in the incidence or progress of some diseases with the occurrence of a GM disorder [7]. Recently, the relationship between the GM and diseases of nervous system has attracted more attention in the medical community, and many studies indicate that the GM can control nervous, endocrine and immune communication via the gut–brain axis and participate in the occurrence as well as the development of nervous system’s disease [8,9,10]. For instance, there is evidence indicating the presence of GM dysbiosis in individuals diagnosed with multiple sclerosis. Studies conducted on animal models of multiple sclerosis have demonstrated that compounds produced by anti-inflammatory bacteria have the potential to mitigate immunological damage [11]. At the same time, a recent study has shown that enteric glia and the microbiota–gut–brain axis can promote inflammatory states, participating in the onset and progression of wrong folding α-synuclein proteins and the start of the early non-motion symptoms of PD [12].

Of course, within the domain of epilepsy, research related to the GM is progressively making headway. The evidence from both animal studies and human cases has also been found, indicating that a dysbiosis in the gut can probably be associated with epilepsy [13,14]. Meanwhile, in a Mendelian randomization analysis, it was also stated that the GM was closely related to epilepsy [15]. Ketogenic diet functions as an alternative therapy of drug-resistant epilepsy. In recent years, it has also been indicated that the ketogenic diet treatment of epilepsy is correlated with alterations in the composition of the GM [16]. For example, using a series of experimental methods including bacterial colony transplantation and antibiotic interference, Olson et al. found that the ketogenic diet had a favorable impact on seizures by regulating the metabolic levels in serum or brain through the GM. In a pilocarpine epilepsy mouse model, 16sRNA sequencing combined with a nuclear magnetic resonance metabolite analysis showed that differential gut microbes were associated with some metabolites maintaining epileptic brain activity [17]. Recently, it has been found in deeper intestinal microscopic mechanisms that the GM metabolites mediate the bax gene to lower neuronal apoptosis via the cGAS/STING axis in epilepsy [18]. Numerous clinical and preclinical investigations have also demonstrated the potential of probiotics [19,20,21] and fecal microbiota transplantation [22] in ameliorating the GM dysbiosis and mitigating seizures through the augmentation of beneficial microbial populations. Briefly, these data all confirm that the GM may be engaged in the pathophysiological process of the occurrence and development of epilepsy.

Recently, a microbiome study based on high-throughput gene sequencing has significantly deepened our understanding of the correlation between the GM and health [23]. Some progress has been achieved in comprehending the intestinal flora of patients with epilepsy. However, it is difficult for a gene sequencing method to directly identify the functional activity of microorganisms and detect the key functional molecules. Thus, the metabolomics of the GM provides clues for investigating the microbial–host interaction mechanisms and becomes a vital complement to gut functional metagenomics [24]. Because the metabolites of the GM need to be delivered to distal target organs through the blood, compared with fecal metabolomics, serum metabolomics can better show the correlations between the intestinal flora and distal organs and pathways [25]. In this pilot study, we were the first to apply an integrated metagenomic and serum metabolomic analysis to comprehensively evaluate the relationship between the GM and serum metabolites in patients with adult epilepsy.

## 2. Methods and Materials

### 2.1. Study Participants and Sample Collection

We recruited patients with epilepsy from the Neurology Department (Epilepsy Clinic) of the Second Hospital of Hebei Medical University, from January 2022 to January 2023. And the approval was received from the Clinical Ethical Committee of the Second Hospital of Hebei Medical University (No. 2022-R002). Patients who satisfied all the following inclusion criteria were involved in this study: (1) the diagnostic criteria for epilepsy of 2017 International League Against Epilepsy; (2) patients’ age ranging from 18 to 60 years; and (3) a single history (epilepsy) with unknown etiology. Exclusion criteria: (1) during the past three months, patients using medications such as antibiotics, nutritional supplements, prebiotics or probiotics, laxatives, antispasmodic or antidiarrhea drugs; (2) smokers or alcoholics; (3) menstruation, pregnant or breastfeeding female patients.

Gender-, age- and ethnicity-matched normal controls were recruited from the Physical Examination Center within the same period. Normal controls conforming to the following criteria were included, containing those aged 18–60 years, those without any medical history, those without any medication history in the past three months and those satisfying the exclusion criteria for epilepsy patients (2), (3). The dietary patterns of patients and normal controls were assessed by 24 h dietary recall [26,27] including energy, protein, fat, vitamins and trace elements.

Finally, we enrolled 22 patients with epilepsy and 10 normal controls. All participants provided written informed consent prior to their involvement in this study. Stool and venous blood samples were obtained from all the enrolled participants on an empty stomach between 6:00 and 7:00 a.m. Stool collection: Participants were required to urinate before defecating. The stool was collected in a sterile stool collection box. Then, fecal samples were collected and processed by clinical technicians and immediately transferred to −80 °C for storage. Venous blood collection: Venous blood was collected by the clinical professional blood collection personnel, stood at room temperature for 60 min and was subjected to centrifugation at 3000 rpm for 15 min at 4 °C, aiming to acquire the serum samples.

### 2.2. Metagenomic Analysis

Metagenomic sequencing involves the random breaking of microbial genomic DNA into small fragments and the subsequent addition of joints at both ends of the fragments for high-throughput sequencing, facilitating in-depth studies at the gene and function levels. Therefore, we chose metagenomic sequencing to analyze intestinal flora [28]. Details are presented in the Supplementary Methods S1.

### 2.3. High-Throughput Targeted Metabolomics

Totally 346 metabolites in the sample were verified by absolute quantification using a liquid chromatography–tandem mass spectrometry (LC-MS/MS)-based targeted metabolomic approach. These metabolites mostly contained amino acids and their derivatives, lipids and their derivatives, bile acids, benzene ring compounds, carbon metabolism compounds, organic acids and their derivatives, indoles and purine nucleotides. The implicated metabolic pathways mainly involved fatty acid metabolism, amino acid metabolism and carbon metabolism. Details are presented in the Supplementary Methods S2.

### 2.4. Metagenomics Data Analysis

R was used to construct column plots of microbial abundance at each level and heatmap on genus level. PCA analysis used R ade4 package, Version 2.15.3. ANOSIM analysis for similarity comparison between groups. Wilcoxon rank-sum tests were employed to find the different species between the groups. *p*-Values < 0.05 thought to be of statistical significance. Microbial species differences were visualized using STAMP software (V2.1.3). To blast unigenes against the KEGG database, we applied DIAMOND software (V0.9.9), predicting the function of the microbe.

### 2.5. Metabolomics Data Analysis

Following sum normalization, the processed data were uploaded into SIMCA-P (version 14.1, Umetrics, Umea, Sweden), in which it was subject to multivariate data analysis, containing 3D Pareto-scaled principal component analysis (3D PCA), partial least-squares discriminant analysis (PLSD-DA) and orthogonal partial least-squares discriminant analysis (OPLS-DA). Variable weight values (VIP) were obtained from the PLSDA analysis. Wilcoxon rank-sum tests were employed to find the different metabolites between the groups. VIP > 1.0 and *p* < 0.05 were chosen to be the differential metabolites. Receiver operating characteristic curve was applied to predict serum metabolic markers. The metabolic pathways of differential metabolites were annotated by the Kyoto Encyclopedia of Genes and Genomes (KEGG) database (https://www.kegg.jp/kegg/pathway.html accessed on 13 June 2022). We acquired the pathways engaged in differential metabolites.

### 2.6. Statistical Analysis

The baseline data analysis was carried out with SPSS statistical software (version 26.0). Data were presented as mean  ±  SD (standard deviation). Continuous variables were tested with the use of the *t*-test and Wilcoxon rank-sum tests. Then, the Spearman correlation was adopted for evaluating the relationship between microorganisms and metabolites. *p*-Values < 0.05 presented statistical significance.

## 3. Results

### 3.1. Participant Characteristics

To investigate the gut microbiome and serum metabolome changes in individuals with epilepsy, we gathered fecal DNA and serum samples from a cross-sectional cohort of 32 subjects for shotgun metagenome sequencing and high-throughput targeted metabolomic analyses, respectively. No obvious differences were found in body mass index, sex or age between the two groups. All participants have a similar dietary structure. We recorded the type and frequency of seizures and whether there was a precursor to the seizure (Table 1).

### 3.2. Characteristics of GM between Patients with Adult Epilepsy and Normal Controls

**Figure 1:**
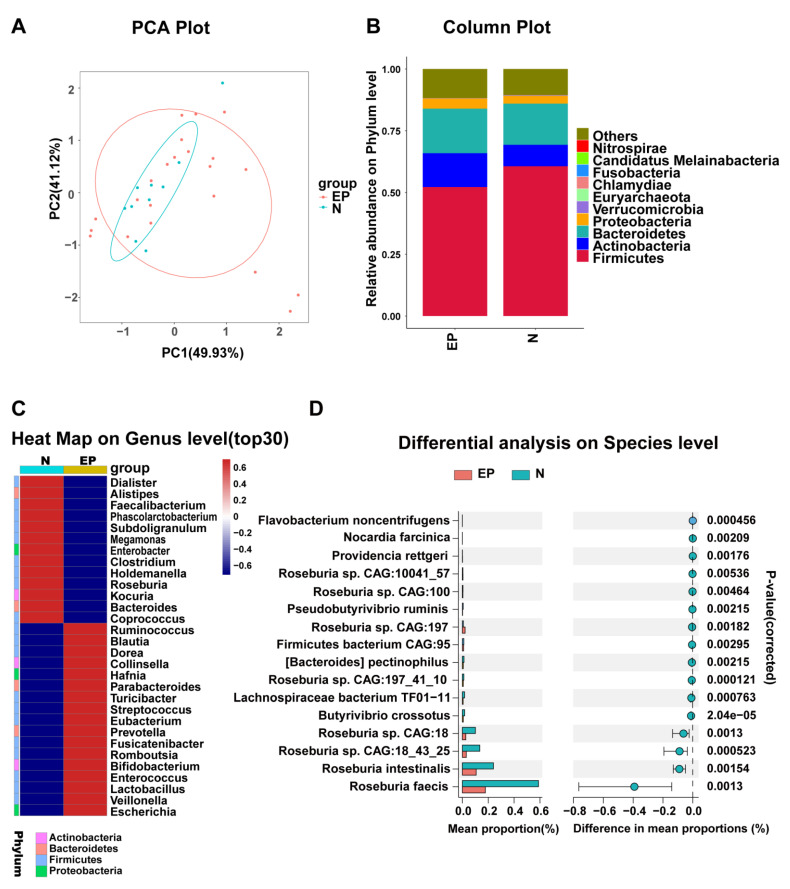
Microbial profile analysis in epilepsy patients and normal controls. (**A**) PCA plot: principal component analysis of two groups. (**B**) Column plot at the phylum level: histogram of species abundance at the phylum level. (**C**) Heat map at the genus level: the genera are presented in rows, and relative abundance is represented by a color gradient. (**D**) Differential analysis at the species level: column chart of top 15 species with significant difference at the species level.

After Illumina sequencing, a total of 11,544 species were predicted, including eukaryotes (0.17%), viruses (1.08%) and procaryotes (98.75%). The analysis of similarities (ANOSIM, R = 0.152, *p* = 0.039) suggests the composition of the gut microbiota of EP differs from that in normal and the overall structure of the community distribution of the two groups can be seen in the principal component analysis (PCA) plot (Figure 1A). Among the eukaryotic microorganisms at the phylum level, the proportion of *Ascomycota* was lower, and that of *Basidiomycota* was notably higher in the EP group compared to the normal group. At the class level, increased levels of *Agaricomycetes* and *Saccharomycetes*, as well as decreased levels *Eurotiomycetes*, were found in the EP group. At the order level, the relative abundance of *Russulales*, *Saccharomycetales* and *Hypocreales* was higher, and that of *Neocallimastigales* and *Eurotiales* was lower in the EP group. At the family level, the EP group exhibited a higher relative abundance of *Bondarzewiaceae* and *Saccharomycetaceae*, and a lower relative abundance of *Neocallimastigaceae*, *Mucoraceae* and *Aspergillaceae*. At the genus level, *Beauveria*, *Anaeromyces*, *Mucor* and *Piromyces* had a lower relative abundance in the EP group, while *Saccharomyces* had a higher relative abundance (Supplementary Figure S1A–E). Among viral microorganisms, we found that, compared with the normal controls, the EP patients had a lower relative abundance of *Myoviridae* (family), *Cba41virus* (genus), *Se1virus* (genus) and *Sp18virus* (genus), as well as a higher relative abundance of *Podoviridae* (family), *Marseilleviridae* (family), *Cbastvirus* (genus), *T7virus* (genus) and *Sfi21dt1virus* (genus) (Supplementary Figure S2A,B). These data show that patients with epilepsy may have diverse profiles of eukaryotes and viruses. Among the prokaryotes with the largest proportion and the highest abundance at the phylum level, the relative abundance of *Actinobacteria*, *Bacteroidetes* and *Proteobacteria* was increased in the patients with EP, whereas that of *Firmicutes* was decreased (Figure 1B, Supplementary Table S1). Based on the hierarchically clustered heat map analysis of the top 30 genera with the highest abundance of the GM at the genus level, the relative abundances of *Dialister*, *Alistipes*, *Faecalibacterium*, *Phascolarctobacterium*, *Subdoligranulum*, *Megamonas*, *Enterobacter*, *Clostridium*, *Holdemanella*, *Roseburia*, *Kocuria*, *Bacteroides* and *Coprococcus* was decreased in the patients with EP (Figure 1C). Compared to the controls, the relative abundances of *Ruminococcus*, *Blautia*, *Dorea*, *Collinsella*, *Hafnia*, *Parabacteroides*, *Turicibacter*, *Streptococcus*, *Eubacterium*, *Prevotella*, *Fusicatenibacter*, *Romboutsia*, *Bifidobacterium*, *Enterococcus*, *Lactobacillus*, *Veillonella* and *Escherichia* was increased in the patients with EP. Our analysis of the metagenomics data allowed for taxonomic identification down to the species level. Thus, a further abundance analysis revealed 335 species that differed between the two groups at the species level. The top 15 microbial strains with obvious differences in abundance are presented in Figure 1D and Supplementary Table S2. For example, the abundance of *Roseburia faecis*, *Roseburia intestinalis*, *Roseburia CAG:18_43_25* and *Roseburia CAG:18* was found to be low in the patients with EP. In overview, there appeared to be differences in the composition of the gut flora between the patients with EP and the controls.

**Figure 2:**
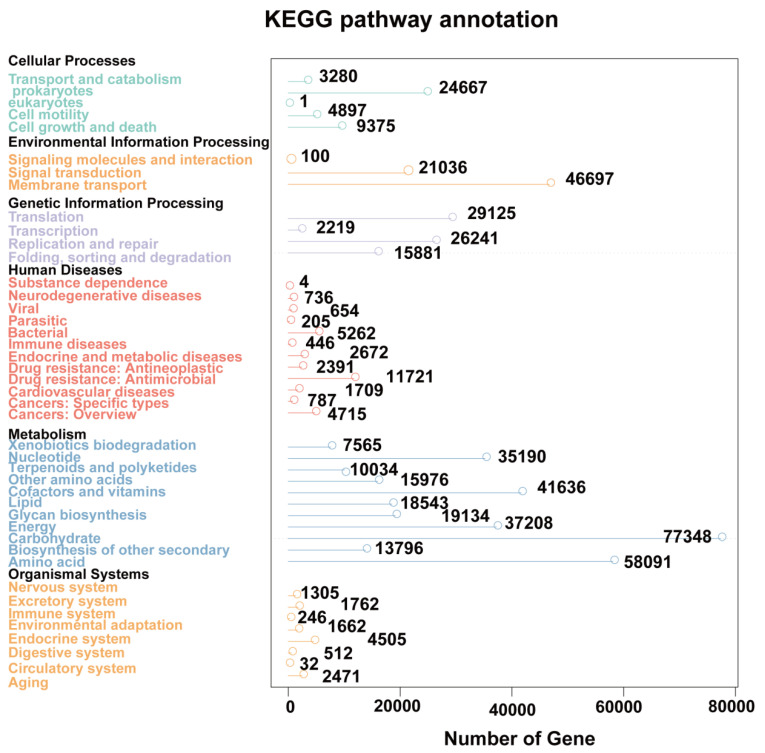
KEGG pathway annotation: functional annotation of the whole unigenes presented as 24 categories of KEGG pathways. The left vertical axis indicates the primary and secondary classification information of KEGG pathway.

Metagenomic studies can reveal potential differences in the gene function between the EP and control groups. In order to predict the gene function of the fecal microbiome through the KEGG pathway library, the DIAMOND software (V0.9.9) was used. We annotated 24 different KEGG pathways (Figure 2).

### 3.3. Characteristics of Serum Metabolites in Patients Suffering from Epilepsy and Normal Controls

Using the UHPLC-MS/MS technique, a total of 179 metabolites were found in both positive and negative ion modes. Regarding quantity, amino acids and their derivatives accounted for 25.7%, lipids and their derivatives occupied 22.9%, and bile acids accounted for 17.9%.

**Figure 3:**
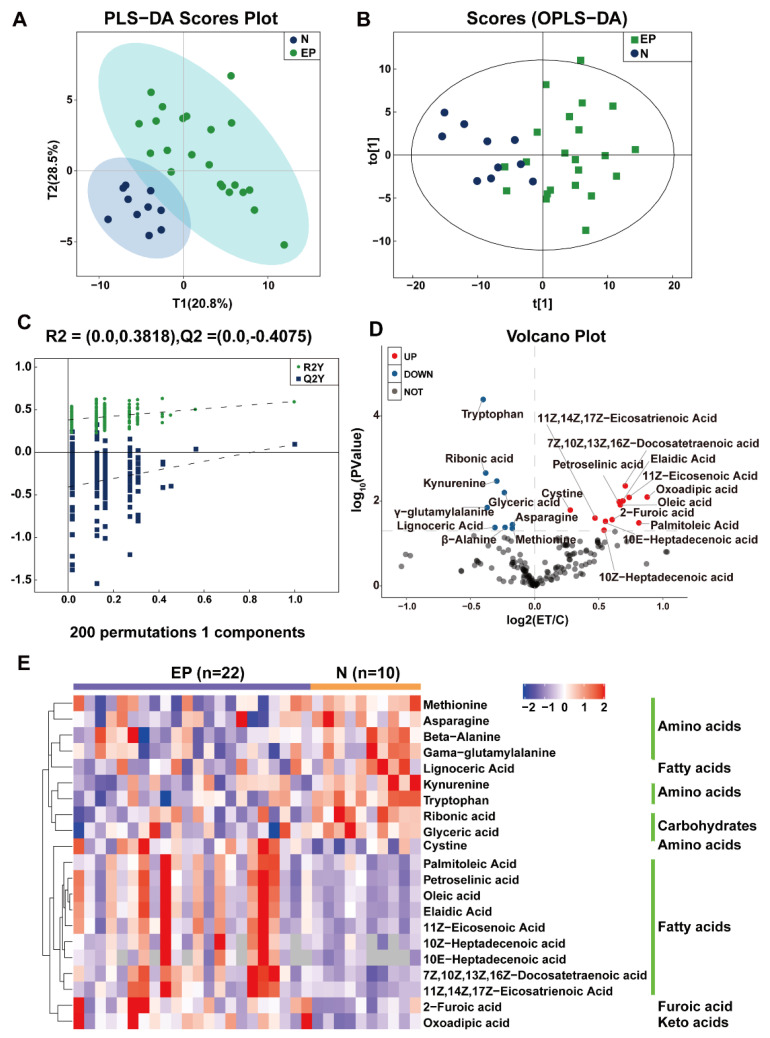
Serum metabolic profile analysis in epilepsy patients and normal controls. (**A**) PLS-DA plots of the serum metabolites from all groups in the positive and negative ion modes. (**B**) OPLS-DA analysis of alterations in the metabolite profile. (**C**) Permutation test of OPLS-DA model. (**D**) Volcano plot of differential metabolites between the control and epilepsy groups; blue: downregulated metabolites; red: upregulated metabolites. (**E**) Cluster heat map showing relative abundance of differential metabolites: from blue (less) to red (more).

**Figure 4:**
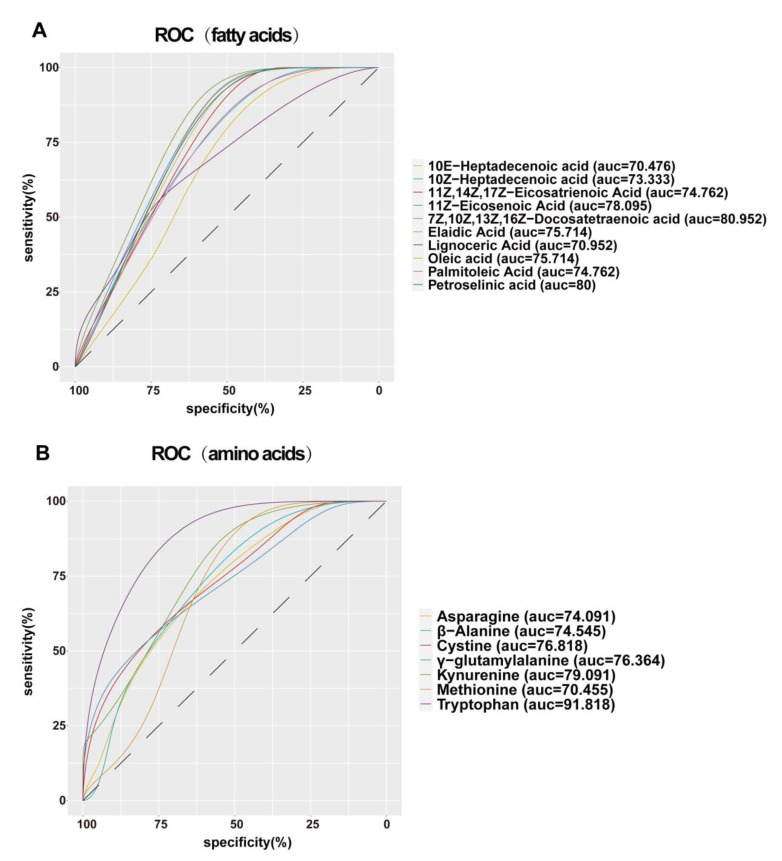
Receiver operator characteristic curves were analysed for the correlation between the specific metabolites and EP.(**A**) Receiver operator characteristic curves of fatty acids. (**B**) Receiver operator characteristic curves of amino acids.

The PLAS-DA and OPLAS-DA models were used for a multidimensional analysis. The score plot of the PLAS-DA model exhibited a clear discrimination between the patients with EP and the controls, with little overlap (Figure 3A). After improving the analytical ability and effectiveness of the model, the OPLAS model (Figure 3B) and model validation (Figure 3C) indicated that there were obvious differences in serum metabolomics between the two groups. Totally 21 significantly differential metabolites were identified, including lipids and their derivatives (10E-heptadecenoic acid, 10Z-heptadecenoic acid, 11Z, 14Z, 17Z-eicosatrienoic acid, 11Z-eicosenoic acid, 7Z, 10Z, 13Z, 16Z-docosatetraenoic acid, elaidic acid, lignoceric acid, oleic acid, palmitoleic acid, petroselinic acid), amino acids and their derivatives (asparagine, beta-alanine, cystine, gamma-glutamylalanine, kynurenine, methionine, tryptophan), carbohydrates (glyceric acid, ribonic acid), 2-furoic acid and oxoadipic acid. The volcano plot shows that the patients with EP have increased concentrations of metabolites related to fatty acids and lower levels of metabolites related to amino acids compared with the corresponding controls (Figure 3D, Supplementary Table S3). To comprehensively and intuitively reveal the expression differences in each metabolite between the patients with EP and the controls, a hierarchical clustering heat map was constructed (Figure 3E). Considering the highly divergent abundance of fatty acids and amino acids in the EP group, further investigations were conducted on these metabolites. Areas under the receiver operating characteristic curve analyzed for the correlation between the specific metabolites and EP are displayed in Figure 4A,B. We can observe that differential serum metabolites have certain diagnostic values. Among them, tryptophan (AUC = 91.81, *p* < 0.0001), kynurenine (AUC = 79.09, *p* < 0.01) and 7Z,10Z,13Z,16Z-Docosatetraenoic acid (AUC = 80.95, *p* < 0.01) could be used as a diagnostic marker for epilepsy.

**Figure 5:**
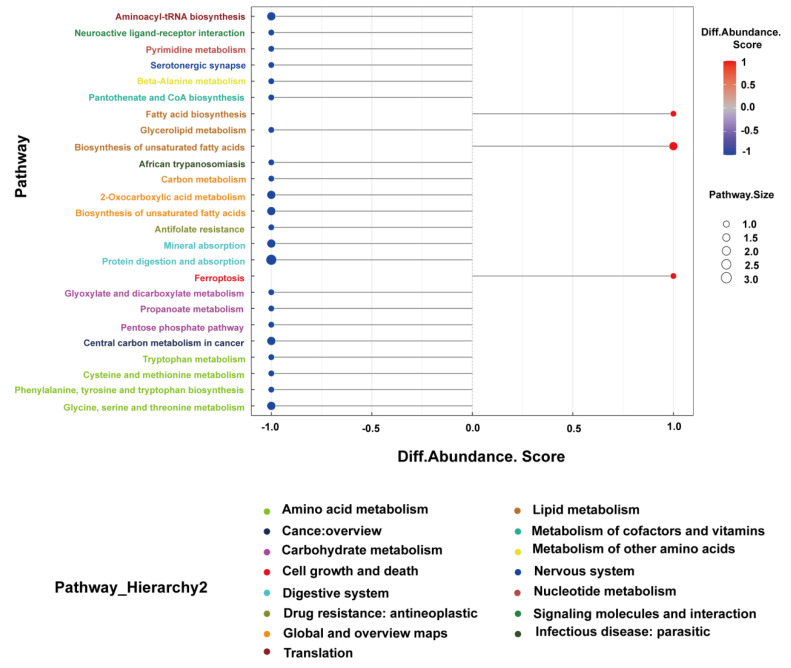
KEGG analysis of differential serum metabolites between epileptic patients and normal controls.

The KEGG database was adopted for a pathway analysis, and serum differential metabolites were indicated to be enriched in tryptophan metabolism; ferroptosis; glycine, serine and threonine metabolism; fatty acid biosynthesis; neuroactive ligand–receptor interaction; serotonergic synapse and other metabolic pathways (Figure 5).

### 3.4. Microbiome–Metabolome Joint Analysis

**Figure 6:**
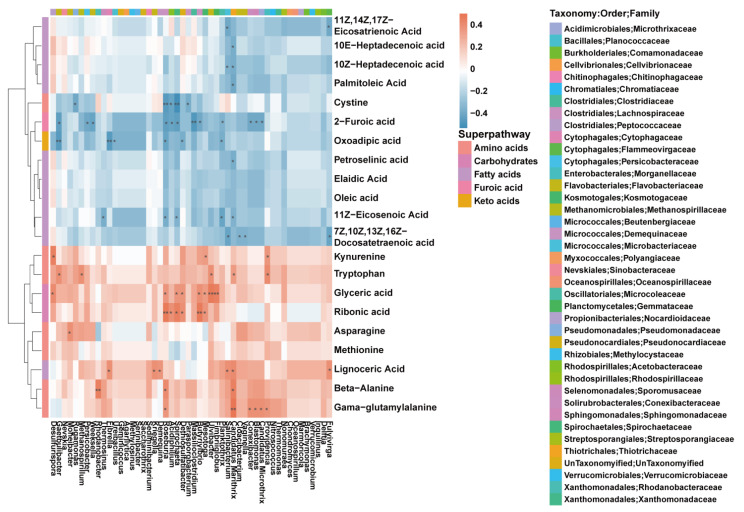
Correlation analysis between 50 differential genera (Wilcox_test *p* < 0.05, top 50) and differential metabolites (* *p*-value < 0.05; ** *p*-value < 0.01).

**Figure 7:**
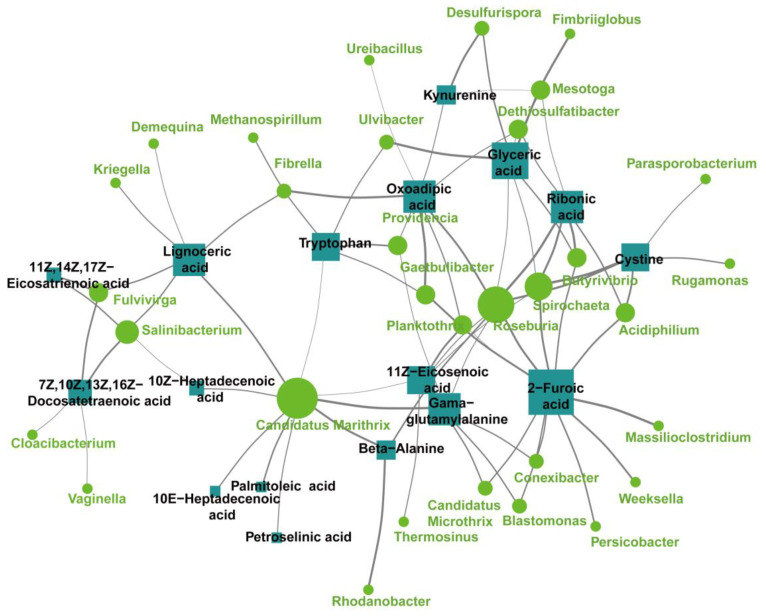
Adult epilepsy-associated networks based on integrated fecal microbiome and serum metabolome (Spearman’s rank correlation analysis, r > 0.3, *p* < 0.05). Squares represent differential serum metabolites, and circles represent differential flora. The size of the boxes and circles indicates the number of associations (bacteria or metabolites). The thickness of the line represents the magnitude of the correlation.

Based on a correlation analysis between the microbiome and metabolome, this study calculated the Spearman’s correlation between the 50 differential genera (Wilcox_test *p* < 0.05, top 50) and the differential metabolites (Figure 6). A statistically significant association was discovered between the differential flora and the differential serum metabolites. For example, the abundance of *Providencia*, *Candidatus Marithrix*, *Ulvibacter*, *Methanospirillum* and *Gaetbulibacter* was positively associated with serum tryptophan levels. Meanwhile, *Providencia*, *Mesotoga* and *Desulfurispora* revealed a positive correlation with serum kynurenine. The abundance of *Fulvivirga*, *Cloacibacterium*, *Vaginella* and *Salinibacterium* was negatively related to serum 7Z,10Z,13Z,16Z−Docosatetraenoic acid levels. To better understand the correlation between the gut microbes and metabolites, the findings were combined to establish a network, aiming to present the correlation between the microbiome and metabolome more intuitively (Figure 7). Based on the obtained results, serum metabolites and related pathways are altered in the patients with epilepsy compared to the normal controls.

## 4. Discussion

The GM is increasingly regarded as a vital contributor to epilepsy. Metabolomics selects all small molecular metabolites of microbial community as the research object. In addition, it can also find the key metabolites of intestinal microorganisms with pathophysiological changes in the host and provide clues for investigating microbial–host interaction mechanisms [29]. The combined application of metagenomics and metabolomics can address the restrictions of the single-omic studies to a great extent and improve the progress in exploring the correlation between the intestinal microbes and diseases [30,31]. In the previous studies of epilepsy, most of the studies were single-omic. Therefore, a combined multiomic analysis was performed in this pilot study.

As part of the present study, stool samples were gathered from epileptic and normal adults to determine changes in the gut macrogenomic profile of the epileptic patients. When compared to the normal controls, the epilepsy patients exhibit distinct microbial profiles in eukaryotes, viruses and prokaryotes. Because the relative abundance of eukaryotes and viruses in the GM is low, they make up a relatively minor portion of it. In light of the current study and other research, we consider that bacterial alterations play a significant role in epilepsy, while eukaryotic and viral alterations cannot be completely ruled out. To clarify the role of eukaryotic and viral participation in the emergence of epilepsy, larger samples and additional research are required. Among the prokaryotes, at the phylum level, *Proteobacteria* and *Actinobacteriota* were notably increased in the patients with epilepsy compared with the controls, conforming to the findings of a recent study [32]. There were also obvious differences between the epilepsy and control groups at the genus and species level. Some of the differential microbiota found in patients with epilepsy are correlated with neurotransmitters. For example, it was reported that *Bacteroides* could digest and metabolize high fat food as well as control the secretion of interleukins-6 and 17 in dendritic cells, a process in strong relationship to seizure severity of epileptic patients [33,34]. *Ruminococcus* was positively related to glutamate and glutamine. In addition, *Alistipes* and *Veillonella* are related to the regulation of inflammation [35]. Alterations in these microbiotas may show a close association with the pathogenic mechanism of epilepsy. To thoroughly understand the function of the GM characteristics of the epilepsy patients, metagenomic sequencing was used in this study. Through the functional annotation analysis of differential unigenes, we discovered that the abundance of the GM in epilepsy patients could influence some physiological active functions. These data suggest that the composition of the GM in epilepsy patients may affect the occurrence of epilepsy by affecting neuroinflammation, signaling pathways and other forms. Regarding the expression of unigenes, six classes of KEGG pathways were identified, with metabolism being the class having the highest number of unigenes.

Herein, we combined metagenomics and metabolomics to show the interaction between the GM and epilepsy. Patients with epilepsy exhibited a significant difference in serum metabolites compared with healthy individuals, with 12 metabolites significantly upregulated and nine metabolites significantly downregulated. Obviously, we found some metabolites that had not been reported in previous studies, including carbohydrates, such as glyceric and ribonic acid, 2-furoic acid and oxoadipic acid. Through the area under the receiver operating characteristic curve, we observed that tryptophan, kynurenine and 7Z,10Z,13Z,16Z-Docosatetraenoic acid was a biomarker to distinguish the epilepsy groups. Tryptophan can be metabolized to kynurenine to regulate neuroendocrine and intestinal immune responses [36]. Kynurenine is found to have neuroprotective properties and competitively hinder ionotropic glutamate receptors at high concentrations [37]. It is widely known that glutamate is the principal excitatory neurotransmitter of the mammalian brain, which can act by modulating ionotropic and metabotropic glutamate receptors. In addition, an imbalance between the excitatory and inhibitory neurotransmission can be found at the core of the pathophysiology of epilepsies. When glutamate receptors were inhibited, cystine uptake was significantly decreased, providing a therapeutic option for seizure suppression [38]. Moreover, kynurenine has been shown to inhibit the N-type Ca2+channels of sympathetic neurons and astrocytes, which can finally result in the suppression of many inflammatory pathways [39]. In this study, serum tryptophan and kynurenine levels were lower in adults with epilepsy compared to healthy individuals. Furthermore, tryptophan was annotated onto the tryptophan metabolism pathway in the KEGG analysis. In general, changes in tryptophan metabolism may have a vital impact on the development of epilepsy.

The combined multiomic analysis, indicated that there was a significant correlation between the differential flora and tryptophan and kynurenine. The abundance of *Providencia*, *Candidatus Marithrix*, *Ulvibacter*, *Methanospirillum* and *Gaetbulibacter* was positively associated with serum tryptophan levels. Meanwhile, *Providencia*, *Mesotoga* and *Desulfurispora* revealed a positive correlation with serum kynurenine. These may be the underlying mechanisms of GM disorders affecting the occurrence of epilepsy following abnormal tryptophan–kynurenine metabolism.

Through the metabolomic sequencing, we found that higher levels of serum 7Z,10Z,13Z,16Z-Docosatetraenoic acid in the patients with epilepsy were annotated onto the ferroptosis pathway in the KEGG analysis. Ferroptosis is a newly found form of regulated cell death providing the nexus between metabolism, redox biology and human health, which has been demonstrated to have a vital impact on various neurological disorders, containing Alzheimer’s disease and Parkinson’s disease [40,41]. The central aspect of ferroptosis refers to the iron-dependent dysregulation of lipid oxidation metabolism. In the accumulation of lipid peroxides in ferroptosis, polyunsaturated fatty acids (PUFAs) are key substances. Adrenic acid (AdA) is one of the polyunsaturated fatty acids and contains diallyl hydrogen atoms, which can easily react with reactive oxygen species (ROS) and cause lipid peroxidation [42]. Recently, there have been few studies on iron death in epilepsy. In animal experiments, it has been discovered that ferroptosis is engaged in seizure genesis in pentylenetetrazole kindling and pilocarpine-induced mouse models [43]. The detailed mechanisms of ferroptosis in the regulation of epileptogenesis have not been clarified. In this study, we observed that different floras in the patients with epilepsy (*Fulvivirga*, *Cloacibacterium*, *Vaginella* and *Salinibacterium*) were negatively related to serum 7Z,10Z,13Z,16Z−Docosatetraenoic acid levels. The mechanism by which ferroptosis affects epileptogenesis may be related to differences in epileptic flora.

Unlike some factors that cannot change but affect the occurrence of epilepsy (such as genetic), at least the composition of the intestinal microflora is likely to change. Improving seizures through the alteration of the gut microbiota appears to be a potential strategy. The method of integrated macrogenomics and metabolomics in this study will undoubtedly offer a better understanding of the molecular mechanisms of microbiota–epilepsy linkage. Our findings indicate that abnormalities in the composition of intestinal flora and imbalance of serum metabolites are related in patients with epilepsy. The identified GM may also be involved in regulating multiple physiological and pathological processes, also offering a theoretical basis for future mechanistic studies of epilepsy. To some extent, the statistics give a support for clinical epilepsy diagnosis and therapy.

However, this pilot study still has the following limitations. The number of subjects was comparatively small, and correlations were not verified. It was not possible to directly link gut microbiome and serum metabolites to the etiology of epilepsy, considering that this is an association study, from which one is unable to draw a causal relationship. In addition, the level of serum metabolites and individual microbiology may be affected by several other factors, including diet and lifestyle. Therefore, we attempted to enhance the standardization of the sampling procedure, aiming to minimize the effect of other interference factors.

In future studies, we will expand the sample size, for instance, using unrelated healthy cohabitants as the controls and collecting information on the diet and lifestyle habits (including physical activity, sleep routine, coexistence with pets and exposure to pollutants) of the participants to reduce some bias. In addition, these results will be further investigated in animal models. Moreover, determining whether the gut microbiota and serum metabolites vary depending on the type of seizure would be an intriguing area of research.

## 5. Conclusions

To conclude, there are significant differences in intestinal flora and serum metabolites in patients with epilepsy compared to normal subjects. Particularly, serum tryptophan, kynurenine and 7Z,10Z,13Z,16Z−Docosatetraenoic acid were available as biomarkers. The imbalance of gut flora in epileptic patients may affect tryptophan metabolism and the ferroptosis pathway. Briefly, the imbalance of gut microbiota, dysregulation of serum metabolites and their interaction may have a vital impact on the pathogenesis of epilepsy. Meanwhile, the specific mechanism needs to be further investigated.

## Figures and Tables

**Table 1:** Characteristics of the participants in this study.

	EP	N	*p*-Value
**Number of patients**	22	10	
**Mean age, years (SD)**	30.4 ± 8.8	28.9 ± 7.6	0.62
**Female, n (%)**	11 (50%)	5 (50%)	
**BMI**	21.04 ± 2.12	21.43 ± 2.01	0.65
**Therapeutic of a drug**			
Yes	10	0	
No	12	0	
**Frequency (seizures per 3 months)**		0	
0–4	10	0	
5–15	2	0	
16–50	5	0	
>50	5	0	
**Epileptic discharges in the EEG**			
Yes	18	0	
No	4	0	
**Aura**			
Yes	13	0	
No	9	0	
**Seizure type**			
Partial seizures	10	0	
Simple partial	4	0	
Complex partial	3	0	
Partial secondary to comprehensive	3	0	
Generalized seizures	12	0	
Absence	3	0	
Tonic	4	0	
Tonic–clonic	5	0	
**Nutrients**			
Energy (kcal)	2431.55 ± 450.32	2309.70 ± 321.41	0.46
Carbohydrate (g)	362.32 ± 35.38	347.30 ± 22.07	0.24
Protein (g)	116.22 ± 19.95	109.80 ± 13.33	0.38
Fat (g)	55.90 ± 3.38	56.82 ± 3.27	0.49
Fiber (g)	22.77 ± 4.29	21.85 ± 3.18	0.56
Cholesterol (mg)	43.42 ± 24.57	459.26 ± 24.99	0.55
Folvite (ug)	528.19 ± 44.79	496.70 ± 37.27	0.07
Vitamin A (ug)	488.00 ± 38.32	513.30 ± 39.05	0.11
Vitamin B1 (mg)	1.48 ± 0.24	1.63 ± 0.19	0.10
Vitamin B2 (mg)	1.26 ± 0.23	1.41 ± 0.22	0.11
Vitamin B6 (mg)	0.41 ± 0.06	0.42 ± 0.05	0.52
Vitamin B12 (ug)	0.53 ± 0.19	0.51 ± 0.18	0.79
Vitamin C (mg)	220.88 ± 22.61	218.65 ± 23.40	0.81
Vitamin D (ug)	2.59 ± 0.44	2.56 ± 0.37	0.80
Vitamin E (mg)	33.56 ± 4.22	33.45 ± 3.57	0.95
Na (mg)	1507.21 ± 125.35	1500.27 ± 99.04	0.88
K (mg)	2307.19 ± 146.52	2327.17 ± 204.62	0.76
Ca (mg)	880.51 ± 81.40	887.69 ± 67.79	0.82
P (mg)	1795.04 ± 92.83	1839.36 ± 61.06	0.19
Mg (mg)	384.92 ± 25.92	365.17 ± 34.61	0.09
Fe (mg)	28.92 ± 2.71	29.33 ± 3.29	0.72
Zn (mg)	17.77 ± 0.77	18.20 ± 0.79	0.17
Cu (mg)	2.32 ± 0.57	2.60 ± 0.38	0.18
Mn (mg)	398.25 ± 38.84	380.17 ± 58.25	0.32
I (mg)	139.09 ± 6.14	136.09 ± 6.11	0.22
Se (mg)	74.29 ± 6.19	75.86 ± 4.86	0.50

Measurement data are expressed as the mean ± SD. EP, epilepsy patients; N, normal controls; BMI, body mass index; NHS, National Hospital Seizure Severity Scale; EEG, electroencephalograph. The results were assessed by a certified neurophysiologist. Aura—a manifestation occurring in the seconds or minutes preceding a seizure such as phantom smell, auditory hallucination, visual hallucination, palpitation, nausea, etc.

## Data Availability

The metagenomic sequencing data for this study have been submitted to the BioProject database, ID: PRJNA941151.

## Supplementary Materials

The following supporting information can be downloaded at https://www.mdpi.com/article/10.3390/microorganisms11112628/s1. Figure S1: The abundance of eukaryotic communities in epilepsy patients at the phylum (A), class (B), order (C), family (D), and genus (E) levels. Figure S2: The abundance of viruses communities in epilepsy patients at the family (A), and genus (B) levels. Table S1: The top 10 gut flora with the largest abundance in each group on phylum level. Table S2: STAMP differential analysis of bacterial populations between the groups at the species level. Table S3: An overview of the metabolomic analysis outcome.
