# Supplementary material for: Integrated Macrogenomics and Metabolomics Explore Alterations and Correlation between Gut Microbiota and Serum Metabolites in Adult Epileptic Patients: A Pilot Study
Source: Microorganisms. 2023 Oct 25;11(11):2628. doi: 10.3390/microorganisms11112628 (PMC10672912; doi:10.3390/microorganisms11112628)
Supplement: Supplementary file 1 [file microorganisms-11-02628-s001.zip › microorganisms-2579124-supplementary.pdf]

**The detailed supplement of method**

**Supplementary methods S1.**

**S1. Metagenomic analysis**

S1.1 Experimental procedure

Total DNA was extracted from a participant's stool using a Stool DNA Kit (TIANGEN, Beijing, China) according to the manufacturer's instructions. For library preparation, DNA samples were fragmented by sonication to a size of 350 bp, then DNA fragments were end-polished, A-tailed, and ligated with the full-length adaptor for Illumina (NEBNext® Ultra™ DNA Library Prep Kit) sequencing with further PCR amplification. PCR products were purified (AMPure XP system) and libraries were analyzed for size distribution by Agilent2100 Bioanalyzer and quantified using real-time PCR (Bio-Rad CFX96). For sequencing, the clustering of the index-coded samples was performed on a cBot Cluster Generation System according to the manufacturer's instructions. After cluster generation, the library preparations were sequenced on an Illumina Novaseq 6000 sequencing platform and 150 bp paired-end reads were generated.

S1.2 Sequencing results pretreatment

Preprocessing the raw data obtained from the Illumina Novaseq 6000 sequencing platform was conducted using Readfq (V8, https://github.com/cjfields/readfq) to acquire the clean data for subsequent analysis. Considering the possibility of host pollution in samples, clean data underwent blast comparison with the host database, using Bowtie 2.2.4 software to filter the reads of host origin. (Bowtie2.2.4, http://bowtie-bio.sourceforge.net/bowtie2/index.shtml)

S1.3 Metagenome assembly

All the reads not used in the forward step of all samples were combined and then software SOAPdenovo (V2.04)/MEGAHIT (v1.0.4-beta) was used for mixed assembly; Scaftigs were obtained by breaking the mixed assembled Scaffolds from the N connection. Fragments shorter than 500 bp were filtered in all Scaftigs, generated from single or mixed assembly, for statistical analysis.

S1.4 Gene prediction and abundance analysis

Using Meta Gene Mark (V2.10, http://topaz.gatech.edu/GeneMark/) software, the Scaftigs (> 500 bp) assembled from both single and mixed assembly, the predicted open reading frame was filtered for length information shorter than 100 nucleotides [1,2] from the predicted results. For predicted open reading frames, CD-HIT software (V4.5.8, http://www.bioinformatics.org/cd-hit) [3] was adopted to remove redundancy and obtain the unique initial gene catalogue. Then, the clean data of each sample was mapped to the initial gene catalogue using Bowtie2.2.4[4,5]. Gene with reads < 2 in each sample were filtered to obtain the gene catalogue (Unigenes) eventually used for subsequently analysis. Finally, statistic the abundance information was determined based on the number of mapped reads and the length of the gene.

S1.5 Taxonomy and functional database annotation

DIAMOND software (V0.9.9, https://github.com/bbuchfink/diamond/)[6] was used to blast the Unigenes to sequences of Bacteria, Fungi, Archaea and viruses extracted from the NR database (Version:2018-01-02, https://www.ncbi.nlm.nih.gov/) of NCBI. For the finally aligned results of each sequence, as each sequence may have multiple aligned results, we selected those with an e value < the smallest e value *10 to take the Lowest Common Ancestor algorithm, which was applied to system classification of the MEGAN software [7] to confirm the species annotation information of sequences. A table containing the number of genes and the abundance information of each sample in each taxonomy hierarchy (kingdom, phylum, class, order, family, genus, species) were obtained based on the Lowest Common Ancestor annotation result and the gene abundance table. The abundance of a species in one sample was equal to the sum of the gene abundance annotated for the species; the gene number of a species in a sample equaled the number of genes whose abundance were nonzero.

1. Nielsen HB, Almeida M, Juncker AS, et al. Identification and assembly of genomes and genetic elements in complex metagenomic samples without using reference genomes. Nat Biotechnol 2014;32(8):822-8.
2. Qin J, Li R, Raes J, Arumugam M, Burgdorf KS, et al. A human gut microbial gene catalogue established by metagenomic sequencing. Nature 2010;464:59-65.
3. Fu L, Niu B, Zhu Z, Wu S, Li W. CD-HIT: accelerated for clustering the next-generation sequencing data. Bioinformatics. 2012;28:3150-2.
4. Qin N, Yang F, Li A, Prifti E, et al. Alterations of the human gut microbiome in liver cirrhosis. Nature 2014;513:59-64.
5. Li J, Jia H, Cai X, Zhong H, et al. MetaHIT Consortium; Bork P, Wang J; MetaHIT Consortium. An integrated catalog of reference genes in the human gut microbiome. Nat Biotechnol 2014 ;32:834-41.
6. Buchfink B, Xie C, Huson DH. Fast and sensitive protein alignment using DIAMOND. Nat Methods 2015;12:59-60.
7. Huson DH, Mitra S, Ruscheweyh HJ, et al. Integrative analysis of environmental sequences using MEGAN4. Genome Res 2011;21:1552-60.

**Supplementary methods S2.**

**S2. High-throughput targeted metabolomics**

S2.1 Metabolite Extractions

To extract metabolites from samples of serum, 400 μL of cold methanol/acetonitrile (1:1, v/v) extraction solvent was added to remove protein and extract the metabolites, then adequately vortexed. For absolute quantification of the metabolites, stock solutions of stable-isotope internal standards were simultaneously added to the extraction solvent. The mixture was collected into a new centrifuge tube, and centrifuged at 14,000 g for 20 min at 4°C, then the supernatant collected and dried in a vacuum centrifuge. For LC-MS analysis, the samples were re-dissolved in 100 μL acetonitrile/water (1:1, v/v) solvent and centrifuged at 14,000 g at 4°C for 20 min, then the supernatant was injected.

S2.2 LC-MS Analysis

Analyses were performed using an UHPLC (1290 Infinity LC, Agilent Technologies) coupled to a QTRAP MS (6500, Sciex) in Shanghai Applied Protein Technology Co., Ltd.. The analytes were separated on HILIC (Waters UPLC BEH Amide column, 2.1 mm × 100 mm, 1.7 μm) and C18 columns (Waters UPLCBEH C18-2.1 × 100 mm, 1.7 μm). The sample was placed at 4°C during the whole analysis process. 6500 QTRAP (AB SCIEX) was performed in positive and negative switch mode. Multiple reaction monitoring was used for mass spectrometry quantitative data acquisition. The multiple reaction monitoring ion pairs are showed in the attached file. Pooled quality control (QC) samples were set in the sample queue to evaluate the stability and repeatability of the system.

S2.3 Data process

MultiQuant or Analyst was used for quantitative data processing. The QCs were processed together with the biological samples. Metabolites in QCs with coefficient of variation less than 30% were denoted as reproducible measurements.

**Supplementary Table S1.** The top 10 gut flora with the largest abundance in each group on phylum level.

| Phylum name | Relative abundance in normal controls | Relative abundance in patients with epilepsy |
|---|---|---|
| Firmicutes | 0.60630642 | 0.52199392 |
| Actinobacteria | 0.08658387 | 0.13697363 |
| Bacteroidetes | 0.16683237 | 0.18056470 |
| Proteobacteria | 0.03164099 | 0.04043603 |
| Verrucomicrobia | 0.00178457 | 0.00077334 |
| Euryarchaeota | 0.00064403 | 5.32E-05 |
| Chlamydiae | 0.00071584 | 0.00097614 |
| Fusobacteria | 7.36E-05 | 0.00017660 |
| Candidatus Melainabacteria | 0.00048153 | 0.00039289 |
| Others | 0.10417114 | 0.11674667 |

**Supplementary Table S2.** STAMP differential analysis of bacterial populations between the groups at the species level.

| species | EP_Mean Rel(%) | N_Mean Rel(%) | Difference between means | P-value | 95% lower CI | 95% upper CI |
|---|---|---|---|---|---|---|
| Roseburia faecis | 0.1777623 | 0.588362 | -0.391588 | 0.001302 | -0.764073 | -0.141043 |
| Roseburia intestinalis | 0.106482 | 0.239969 | -0.090185 | 0.001544 | -0.131031 | -0.049076 |
| Roseburia sp. CAG:18_43_25 | 0.030389 | 0.134759 | -0.088991 | 0.000523 | -0.193601 | -0.036792 |
| Roseburia sp. CAG:18 | 0.026377 | 0.100742 | -0.062562 | 0.001302 | -0.135463 | -0.024793 |
| Butyrivibrio crossotus | 0.007165 | 0.017402 | -0.011650 | 2.04E-05 | -0.015245 | -0.006594 |
| Lachnospiraceae bacterium TF01-11 | 0.005922 | 0.017114 | -0.009638 | 0.000763 | -0.015364 | -0.004094 |
| Roseburia sp. CAG:197_41_10 | 0.007075 | 0.011279 | -0.007094 | 0.000121 | -0.012541 | -0.002653 |
| [Bacteroides] pectinophilus | 0.007176 | 0.012863 | -0.006121 | 0.002149 | -0.009466 | -0.002640 |
| Firmicutes bacterium CAG:95 | 0.009398 | 0.011148 | -0.004788 | 0.002951 | -0.008141 | -0.001516 |
| Roseburia sp. CAG:197 | 0.021185 | 0.007431 | -0.004607 | 0.001824 | -0.007188 | -0.001951 |
| Pseudobutyrivibrio ruminis | 0.001843 | 0.006236 | -0.002337 | 0.002149 | -0.004296 | -0.001015 |
| Roseburia sp. CAG:100 | 0.003947 | 0.004679 | -0.001874 | 0.004639 | -0.002957 | -0.000788 |
| Roseburia sp. CAG:10041_57 | 0.004548 | 0.004052 | -0.001827 | 0.005362 | -0.003171 | -0.000500 |
| Providencia rettgeri | 0.000832 | 0.000404 | -0.000185 | 0.001756 | -0.000441 | -7.16E-05 |
| Nocardia farcinica | 0 | 0.000051 | -8.51E-05 | 0.002089 | -6.94E-05 | 0 |

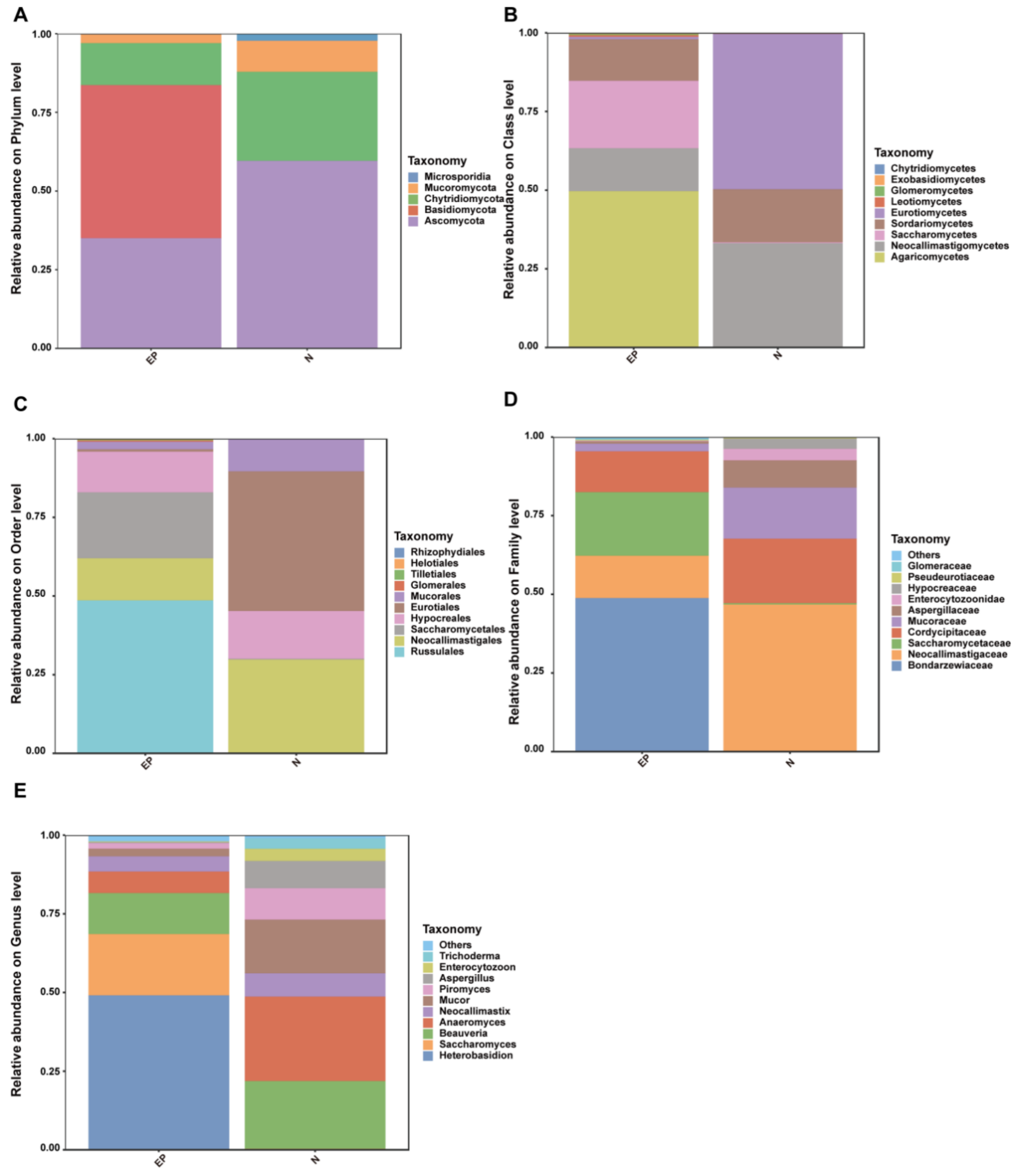


**Supplementary Figure S1.** The abundance of eukaryotic communities in epilepsy patients at the phylum (A), class (B), order (C), family (D), and genus (E) levels.

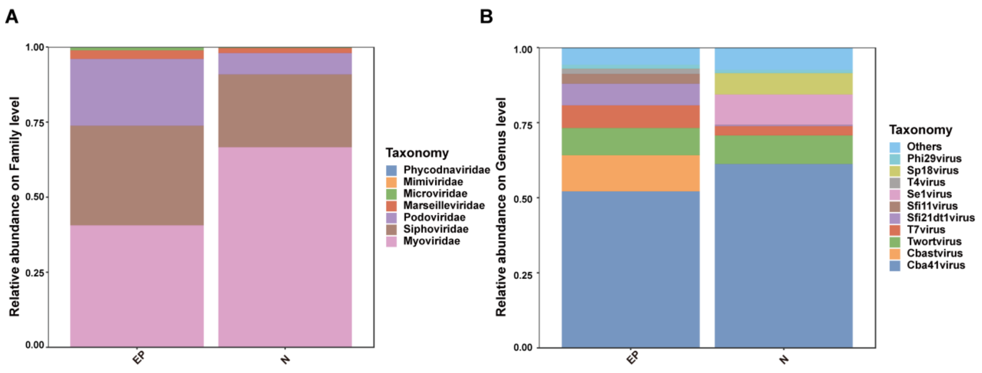


**Supplementary Figure S2.** The abundance of viruses communities in epilepsy patients at the family (A), and genus (B)levels.

**Supplementary Table S3.** An overview of the metabolomic analysis outcome.

| | | |
|---|---|---|
| Epileptic patients vs Normal controls | 179 | amino acids and their derivatives accounted for 25.7%, lipids and their derivatives occupied 22.9%, and bile acids accounted for 17.9% |

**Serum differential metabolites**

| **Name** | **Class** | **Foldchange** | **pvalue** |
|---|---|---|---|
| 10E-Heptadecenoic acid | Fatty acids | 1.466107729 | 0.030036481 |
| 10Z-Heptadecenoic acid | Fatty acids | 1.455198774 | 0.048485219 |
| 11Z,14Z,17Z-Eicosatrienoic Acid | Fatty acids | 1.385919482 | 0.025153048 |
| 11Z-Eicosenoic Acid | Fatty acids | 1.66645794 | 0.008213937 |
| 2-Furoic acid | Furoic acid | 1.520111901 | 0.027457927 |
| 7Z,10Z,13Z,16Z-Docosatetraenoic acid | Fatty acids | 1.63224567 | 0.004419807 |
| Asparagine | Amino acids | 0.887036696 | 0.035906721 |
| Beta-Alanine | Amino acids | 0.852283414 | 0.041615514 |
| Cystine | Amino acids | 1.212246679 | 0.016297136 |
| Elaidic Acid | Fatty acids | 1.611798657 | 0.009907898 |
| Gama-glutamylalanine | Amino acids | 0.773775403 | 0.01422619 |
| Glyceric acid | Carbohydrates | 0.849017823 | 0.006330989 |
| Kynurenine | Amino acids | 0.815099109 | 0.003400228 |
| Lignoceric Acid | Fatty acids | 0.806846508 | 0.041914392 |
| Methionine | Amino acids | 0.886094457 | 0.043876598 |
| Oleic acid | Fatty acids | 1.588997149 | 0.01231258 |
| Oxoadipic acid | Keto acids | 1.837210554 | 0.008072284 |
| Palmitoleic Acid | Fatty acids | 1.75637843 | 0.032708483 |
| Petroselinic acid | Fatty acids | 1.579018116 | 0.010435385 |
| Ribonic acid | Carbohydrates | 0.767521672 | 0.002197188 |
| Tryptophan | Amino acids | 0.75719594 | 4.11096E-05 |
